# Sex Is Always Well Worth Its Two-Fold Cost

**DOI:** 10.1371/journal.pone.0006012

**Published:** 2009-07-07

**Authors:** Alexander Feigel, Avraham Englander, Assaf Engel

**Affiliations:** Soreq NRC, Yavne, Israel; University of Oxford, United Kingdom

## Abstract

Sex is considered as an evolutionary paradox, since its positive contribution to Darwinian fitness remains unverified for some species. Defenses against unpredictable threats (parasites, fluctuating environment and deleterious mutations) are indeed significantly improved by wider genetic variability and by positive epistasis gained by sexual reproduction. The corresponding evolutionary advantages, however, do not overcome universally the barrier of the two-fold cost for sharing half of one's offspring genome with another member of the population. Here we show that sexual reproduction emerges and is maintained even when its Darwinian fitness is twice as low as the fitness of asexuals. We also show that more than two sexes (inheritance of genetic material from three or even more parents) are always evolutionary unstable. Our approach generalizes the evolutionary game theory to analyze species whose members are able to sense the sexual state of their conspecifics and to adapt their own sex consequently, either by switching or by taxis towards the highest concentration of the complementary sex. The widespread emergence and maintenance of sex follows therefore from its co-evolution with the even more widespread environmental sensing abilities.

## Introduction

All modern sexual species emerged from asexual ancestors. Some populations have experienced multiple transitions between sexual and asexual reproductive modes [1], others are capable of changing repeatedly either their sex or mode of reproduction in the course of a single generation [2]. One should explain these transitions and the subsequent maintenance of the reproductive mode on the basis of Darwinian natural selection, finding evolutionary advantages for asexual individuals to differentiate into egg-producing females and sperm-producing males or vice versa.

A paradox emerges by assuming that sexual reproduction increases the Darwinian fitness of a population. Sex implies universally a two-fold fitness cost [3], [4]. A sexual individual shares half of one's offspring genome, while an asexual one generates an almost exact replica of itself. Sexual reproduction transfers thus only half of the individual's genes to the next generation, when compared to parthenogenesis. This results in a two-fold increase in the cost of sex (or of males), assuming no other advantages or shortcomings for the existence of males in a population. This high basic cost causes difficulties to confirm generally that sexual reproduction increases the Darwinian fitness.

The advantages of sexual reproduction stem from quite various roots [5]. For instance, sex increases genetic variability by recombination of the parental chromosomes [6], [7]. It makes a population more resistant against many unpredictable threats, such as deleterious mutations [8], [9], parasites [10], [11], a fluctuating environment [6], [12], [13] or competing groups [14]. It also optimizes the evolutionary search for the best gene combinations in a single individual (epistasis [15], [16]).

Quantitative comparison between costs and advantages of sex can lead to counterintuitive results. For instance, one may estimate the number of deleterious mutation per generation required to make sexual diversity worth its two-fold cost [8]. The derived prediction of one deleterious mutation per generation seems unnaturally high in the light of relevant experimental results [17]. Other qualitative contradictions are discussed in many reviews questioning our understanding of both sexual [9], [18] and asexual [1], [19] species.

The paradox of sex can be solved either by presenting a strong and ubiquitous advantage for sex applicable to all sexual species or by showing how it can evolve despite the reduction to the Darwinian fitness. In this Article we demonstrate the later, showing that sex can maintain its stability even at twice the cost of parthogenesis. According to our results, even a minor sex-promoting effect such as a small amount of deleterious mutations may explain the maintenance and emergence of sexual reproduction, since the requirement for an absolute increase of Darwinian fitness is eliminated in this context. We show that this stability can be achieved through elementary mechanisms of sensing the sexual composition of the environment. Such an ability promotes assortative, instead of random, encounters between complementary sexes by adjustment of the individual's sex or location correspondingly to the environment.

Our proposal for evolution of sex is analogous to evolution of social phenomena, such as cooperation (for a recent review see [20] and references therein). For instance, stability of cooperation may be achieved by preferential interaction between individuals that are willing to cooperate and are able to recognize other cooperators, implying that interactions in such a population cease to be random. Assortative encounters increase and maintain cooperation, though do not necessarily lead to an absolute gain in average Darwinian fitness of the population. Reduction of fitness is common in social models, e.g. evolutionary stable defection in the case of Prisoner's Dilemma [21].

## Results

To discuss the emergence and maintenance of sex, we assume its onset in the development of assortative interactions between complementary sexes, as a consequence of evolving abilities to sense the sexual distribution of the near environment and to optimize the individual's state correspondingly [22], [23]. These skills remain in modern species: attraction (similar to chemotaxis) between different sexes is ubiquitous in all animals [24], [25] and some species, even vertebrates, are able to change sex as a function of their environment [2].

Consider a population composed of organisms possessing either of two sexes *F* or *M*. In an asexual population each individual is in the *F* state. A sexual population corresponds to individuals who are always in the sexual state that is complementary to the average sex of the environment: *F* in a *M* environment or *M* in a *F* environment. We assume the emergence of mutants *M*, having, in addition to self-reproduction (albeit lower than their hosts *F*), also an initially limited ability to pass on their genetic material to the *F* environment.

The development of sexual reproduction is governed by its evolutionary payoffs 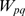 of sex *p* in environment of sex *q* 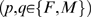:
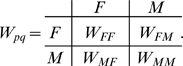
(1)These payoffs include all relevant biological causes contributing to the cost and benefits of different reproduction mechanisms. Payoffs are in general asymmetric, 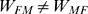 corresponding to unequal costs for males and females. The Darwinian fitness *G* of an individual can be associated with its mean payoff from all possible situations (*F* in *F*, *F* in *M*, *M* in *F* and *M* in *M*). It is determined, therefore, by the individual probabilities to have a specific sex under particular circumstances. These probabilities are defined by the individual characteristics (phenotypes), such as specific sensor, sex taxis or sex switch skills. Evolution proceeds by generations; the existing phenotypes in a population are replaced by favorable mutants possessing greater fitness due to their better abilities. This process culminates in an evolutionary stable population where no mutants can outperform the host phenotype.

The evolutionary stable state of a population is determined by the values of the payoffs 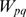. We assume that 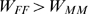, indicating that initial asexual populations were composed of individuals *F* only. The fitness in an asexual population is 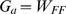. The average fitness in a sexual population 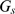 is given by 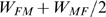, since an individual is either *F* in environment of *M* or *M* in environment of *F*. The cost of sex corresponds to the ratio of average fitness in asexual and sexual population:
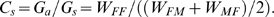
(2)One can analyze, therefore, the evolutionary stability of a sexual populations as a function of cost of sex 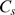.

In natural populations, the cost of sex is
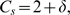
(3) where *δ*indicates deviations from the exact two-fold value. For instance, advantages of sex, such as greater diversity, may reduce 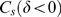. On the other hand, sex may possess additional shortcomings, e.g slower replication mechanisms (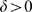). Sexual reproduction is fitter than the asexual one if 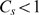. Emergence and maintenance of sex is considered as a paradox, since the cost of sex seems to be 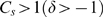 at least in the case of some sexual species.

In this model a phenotype of an individual *h* is described by its conditional probabilities 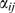 to possess sex *i* in environment of sex *j*. For two sexes *F* and *M*, it comprises two independent evolvable parameters 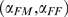 corresponding to the conditional probabilities to possess sex *F* in a *M*- and a *F*-environment respectively. They define the entire set of the conditional probabilities 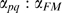, 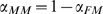, 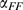 and 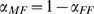. This formalism follows previous works describing behavior by conditional probabilities [26]–[30] and allows accounting for any sex determining mechanisms [31].

To determine whether a population composed of identical individuals 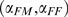 is sexual or asexual, as well as to evaluate its Darwinian fitness, one should determine the corresponding unconditional probabilities 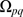 to possess sex *p* in environment *q*. According to the definition of 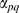 as a conditional probability to possess sex *p* in environment *q*, 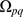 equals 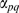 multiplied by the unconditional probability 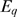 to be in environment *q*:
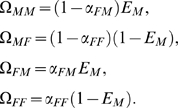
(4) Assuming mean-field conditions in a population (properties of the environment are defined by the average value of individual properties), the probability to be in a specific environment matches the individual unconditional probability to possess the corresponding sex:
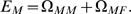
(5) Eqs. (4) and (5) result in:
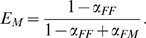
(6) The statistics 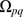 can be expressed, therefore, through 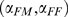 only.

**Figure 1:**
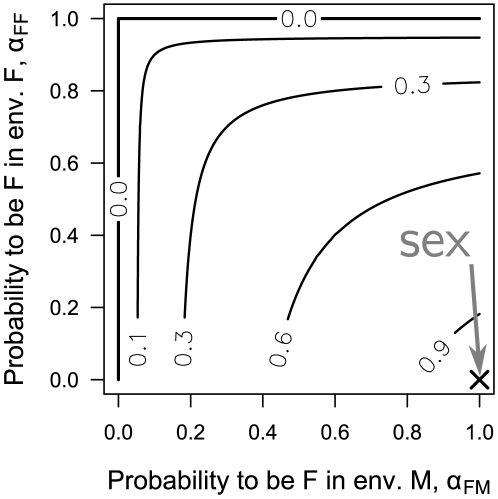
Evolution of sexual reproduction as development of sex switching and sensing abilities. Contour plot of *R* (probability to possess sex complementary to the environment) in populations with varying individual switch and sensor characteristics. The switch/sensor complex is described by 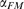 and 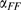, representing conditional probabilities to possess sex *F* in *M* and *F* environments respectively. The single point × denotes a fully developed sexual population (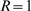) while there exist multiple possibilities for asexual populations (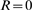). Evolution is equivalent to the motion of a point, denoting a population, from an asexual state to the sexual endpoint. Specific evolutionary mechanisms correspond to different evolutionary pathways.

A population is sexual when it is composed of individuals with 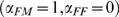 phenotype. This leads to 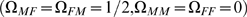 meaning that these individuals always possess sex that is complementary to the environment. A population will be asexual when it is composed of individuals with 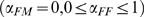 or with 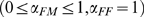, leading to 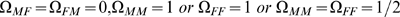 respectively. The sexual state of a population will be characterized by *R*, the probability to be in the sex opposite to the environment: 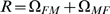. A population is sexual when 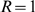, and asexual for 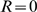 (see Fig. 1).

**Figure 2:**
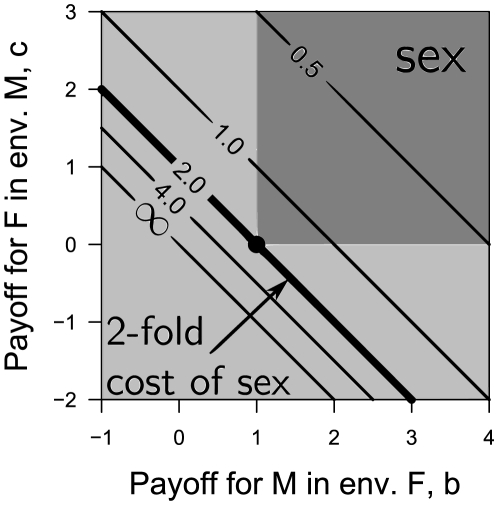
Evolutionary stability of sexual population for different values of cost of sex. The space of evolutionary payoffs, separated into sexual (dark grey) and asexual (light grey) evolutionary stable regions, superimposed on a contour plot of the cost of sex (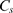). Many previous works suggested that sex is stable only if its fitness is higher than the parthenogenetic one (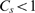 region). We demonstrate that the sensor/switch abilities make sex evolutionary stable for up to two-fold cost (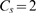). No stability is possible for 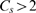.

The evolutionary stability of sex as a function of its cost 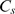 is presented in Fig. 2 (for derivation see “Evolutionary stability of sex”). For the sake of simplicity, we reduce the payoff table (1) to a two parameter form (see “Derivation of normalization”):
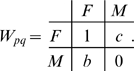
(7) The cost of sex (2) becomes:
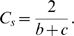
(8) The stability of sexual population and the corresponding cost of sex as a contour plot are shown for values of payoffs *b* and *c*. It should be stressed that transition from (2) to (8) is valid only for 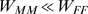, which applies probably to all sexual species. Greater values of 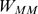 increase the effective cost of sex at each point of the payoff space 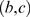.

## Discussion

Sexual populations 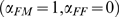 can be evolutionary stable at up to a two-fold cost 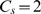, see Fig. 2. At this cost all additional benefits and shortcomings of sex equilibrate each other (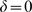, see eq. (3)). This result eliminates the assumption, adopted previously, that sex remains stable only if its benefits overcome its shortcomings 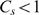 (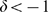). It also explains a peculiar equilibrium between sexual and asexual populations in nature: small deviations from the two-fold cost 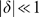 may lead to either sexual or asexual development, corresponding to species that have developed and lost sex during their evolution. It is interesting to note that for the case where only equal parental contribution to the offspring's genome accounts for one's fitness (corresponding to 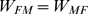, 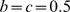 and 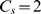) sex remains unstable. The stability can be achieved either through additional benefits (such as diversity, etc.) which reduce the cost of sex or by a certain asymmetry in male and female payoffs (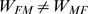), while 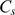 remains unchanged.

The maintenance of sexual reproduction, despite a more beneficial parthenogenesis, may be explained briefly as follows. With developed sensing abilities, the individual choice to spend more time as *F* for the sake of beneficial 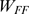 payoffs leads to *F* vs. *M* encounters (with corresponding payoff 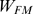), since other members of the population are able to sense and exploit this behavior by adjusting their reproductive mode to *M*.

Three and more sexes can not be evolutionary stable. We define multiple sexes as an equal sharing of the offspring's genome between more than two parent organisms (see previous treatments of this problem [4], [32]). To show this, we assume a population to be sexual if its members, when placed in a sexually pure environment, choose with equal probability a sex that is different from the environment. This definition is an extrapolation of the predisposition to be a male in a female environment and vice versa to the case of hypothetical multi-parental reproduction mechanisms(it does not consider other mechanisms that are considered as multi-sexual though include explicit requirements for two parents, such as reproduction of acellular slime molds [33], fungus *Schizophyllum* and some social insects [34]). Our approach leads to fractional values of the parameters 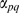 for more than two sexes, causing the corresponding populations to be evolutionary unstable (for instance in the case of three sexes: 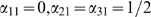) (see “Evolutionary stability of sex” and “Evolutionary stability for more than two sexes”).

An experimental verification of the presented results seems to be possible following the approach of a recent experimental work [35]. It has shown the feasibility to measure the reproductive success of males and females in different environments. Moreover it indicates a possible negative influence exerted by competing males on the reproductive success of asexual females (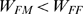 or 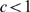). To determine payoffs (1) and the corresponding cost of sex (2) one should measure the reproductive success of each sex in every possible environment (*F* in *F*, *F* in *M*, *M* in *F* and *M* in *M*).

This work suggests a rationale for maintenance of sex, providing a universally applicable reason for overcoming the two-fold cost of sex barrier. It explains the widespread phenomenon of sexual reproduction by its link to even more frequently occurring sensing abilities. It allows subsuming existing and future explanations in a framework that decouples the specific mechanisms dealing with emergence and maintenance of sex from the two-fold cost issue. The results are based on a novel approach to incorporate communication in evolutionary game theory, which can be extended to a general analysis of evolution of information exchange and intelligence [36].

## Analysis

### Evolutionary stability of sex

Consider a population composed of a host *h*, characterized by 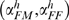 that is challenged by a mutant *m*, characterized by 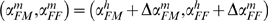. The evolutionary stability of such a population requires that no mutant is fitter than the host:
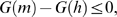
(9) where 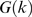 is the fitness of individual *k*.

The fitness is determined by the individual probabilities 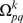 to possess sex *p* in environment *q*, and by the corresponding payoffs 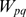:
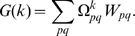
(10) Following eqs. (4) and (6):
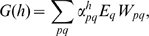
(11) and
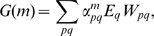
(12) where 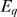 (6) is defined solely by the host's values of 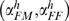 since we assume that the amount of mutants is small.

**Figure 3:**
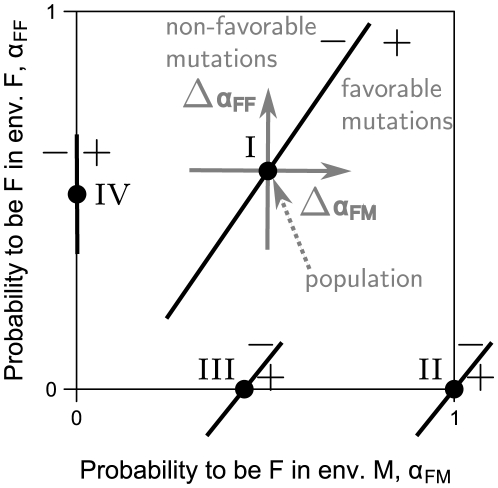
Evolutionary stability of sexual reproduction. The equation for evolutionary stability (13) defines positive and negative semi-planes, corresponding to favorable and non-favorable mutations, dependent on payoffs *b* and *c*. A population with arbitrary 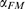 and 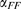 (I) will always dispose of a positive region, precluding so an evolutionary stable solution. A sexual population (II) is evolutionary stable since no positive direction is available. The population on the 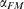 (III) and 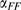 (IV) edges are also unstable. If we assume opposite signs for the semi-planes as consequence of different payoff values, the asexual populations (positioned on the edges 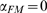 or 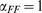) become stable, while the sexual population becomes unstable.

Following eqs. (11), (12) and (7), the condition for evolutionary stability (9) becomes:
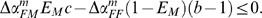
(13) This expression divides, for each specific population (a point in phenotype space 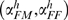), the phenotype space into two semi-planes corresponding to favorable and non-favorable mutations (see Fig. 3).

According to (13), a sexual population 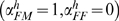 is evolutionary stable for 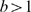 and 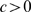. For other payoff values, a population converges to asexual states with 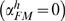 or 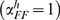. As shown in Fig. 3, the linear properties of (13) prevent formation of stable points with fractional values of 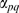 for sexual populations (unless sex *q* is not present, 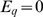 and the corresponding values 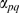 are irrelevant). This remains valid for more than two sexes (see “Evolutionary stability for more than two sexes” for a rigorous proof).

### Derivation of normalization

Reduction of the payoff table:
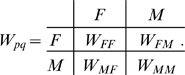
(14) to its two parameters form (7) requires two transformations:
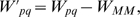
(15) and
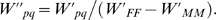
(16) Consequently, the parameters *b* and *c* in (7) are:
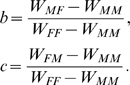
(17)


The transformations (15) and (15) do not affect the stability condition (9). Taking into account (11), (9) becomes 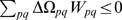:
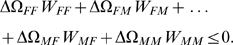
(18) where 
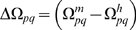
.

Applying the first transformation (15) to (18) results in:
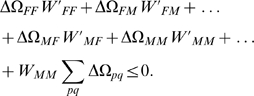
(19) The last term in the left part vanishes 
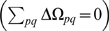
 preserving the form of condition (10). The second transformation (16) converts (19) into 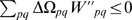.

Expressions for cost of sex (8) and the ratio of fitness in asexual and sexual population 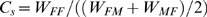 are identical only in case 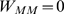. Otherwise, using (17):
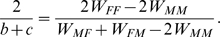
(20) Finite positive values of 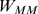, therefore, increase effective cost of sex:
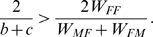
(21)


### Evolutionary stability for more than two sexes

In case of a population with more than two sexes, the corresponding values of 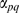 are fractional and located on the edges (rather than nodes) of the phenotype space(see Fig. 3). Such populations can not be evolutionary stable, since, in general, the phenotype space 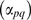 is a *K* dimensions cube, while evolutionary favorable and unfavorable mutations are defined by a 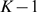 dimensional surface (e.g. see (13)). For instance, the only possibility to confine a point with a line (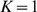) on the edge of a square (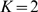) is when the line is parallel to the edge (see Fig. 3, point IV). This occurs if the corresponding sex *q* is not present in the population (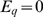). The same reasoning applies to multiple sexes, with *K* dimensional cube and 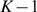 dimensional constraint for evolutionary stability.

A rigorous proof is as follows. The evolutionary stability of specific value of 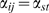 requires 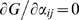 at 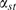 in case 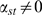 and 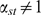. In case of multiple sexes, the expression for fitness remains identical to (11):
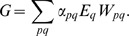
(22) Taking into account that 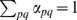, eq. (22) may be rewritten as:
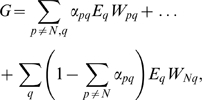
(23) where 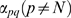 are independent parameters. Consequently:
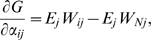
(24) This expression vanishes for 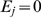 (sex *j* is not present in the population) or for 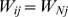 (states *i* and *N* are degenerate in environment *j*).

### Comparison with model games

In the evolutionary game theory, the payoffs (7) are separated into standard model games. According to this work, the games of Leader 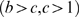, Battle of the Sexes 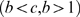 and Chicken 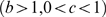 lead to development of sexual reproduction, while Prisoner's dilemma 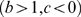 promotes asexual populations.
